# Supplementary material for: A cost analysis comparing seasonal malaria chemoprevention with and without Vitamin A supplementation among under-5 children in Nigeria
Source: PLoS One. 2025 Oct 8;20(10):e0315655. doi: 10.1371/journal.pone.0315655 (PMC12507290; doi:10.1371/journal.pone.0315655)
Supplement: S2 Data — (DOCX) [file pone.0315655.s002.docx]

# ANNEX 1: Main cost categories and associated cost subcategories

| **Main Cost category** | **Cost Subcategory** |
| --- | --- |
| Distribution remuneration | CDD/LM/TA   - Communication - Distribution allowance - Communication allowance |
| Supplies | Data Tool  Procurement of Visibility material |
| Management | Level of effort on Salaries of Country & Field Office staff |
|  | Country Office Support and inventory  Project/technical management support |
| Planning Meetings | Feeding, Transport, DSA, and Accommodation   - Community Leaders/ LGA stake holders Advocacy visit - Health Facility Assessment - LGA Entry meeting - LGA Level finalization of micro-plan - LGA level meeting - Personnel selection - State Level finalization of micro-plan - State level meeting |
| Training | Feeding, transport DSA, trainer fees, allowances   - Community Volunteers (CDD/LM/TA) - Health Facility Workers/ - State level training of trainers - National training of trainers |
| Drugs | SPAQ   - SPAQ1&2   Vitamin A   - Vitamin A 100,000 IU - Vitamin A 200,000 IU |
| Supervision | Feeding, transport DSA, allowances   - Health facility workers - LGA/State/National team |
| Other | Car hire for Last Mile Distribution  Media Operating costs   - Radio jingles   Personal protective Equipment   - Facemask - Hand sanitizer etc.   Storage/warehouse for Vitamin A and/or SMC |
