# Supplementary figures and images for: A cost analysis comparing seasonal malaria chemoprevention with and without Vitamin A supplementation among under-5 children in Nigeria
Source: PLoS One. 2025 Oct 8;20(10):e0315655. doi: 10.1371/journal.pone.0315655 (PMC12507290; doi:10.1371/journal.pone.0315655)

# S3 Annex 2

Annex 2: Sensitivity analyses


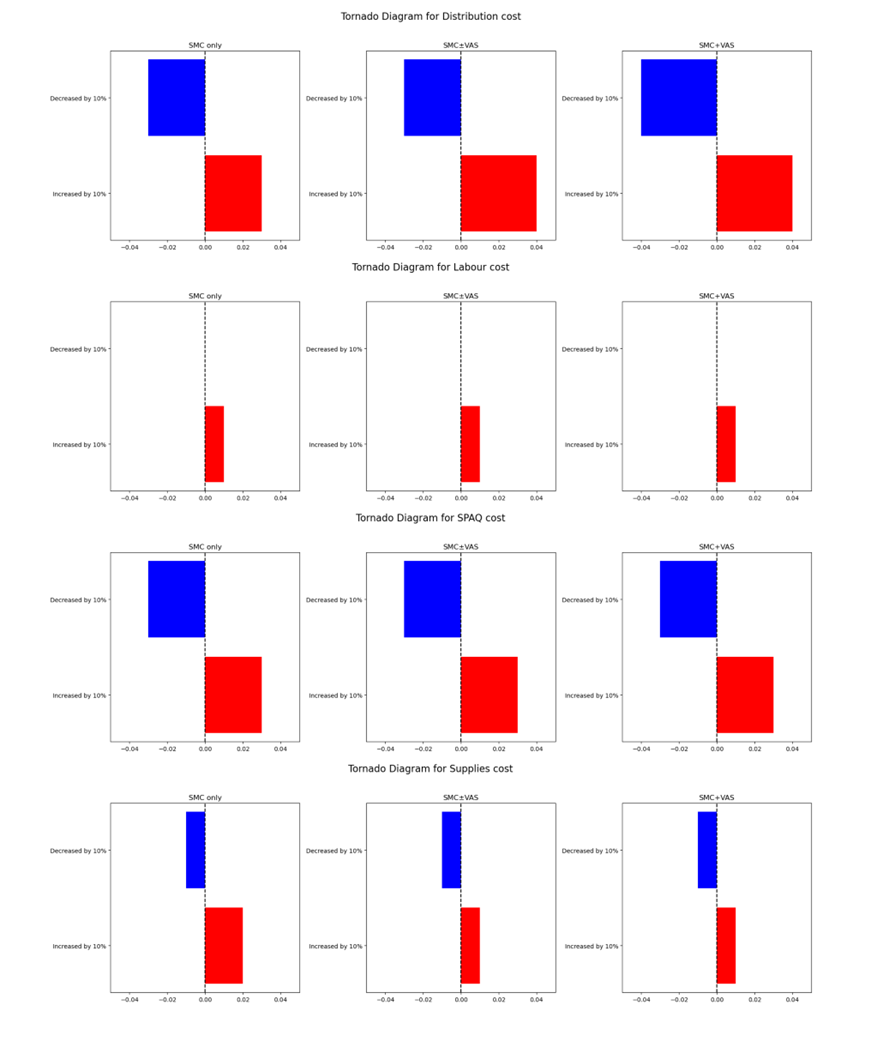

Supplement: S3 Data — (DOCX) [file pone.0315655.s003.docx]
