# Supplementary material for: A cost analysis comparing seasonal malaria chemoprevention with and without Vitamin A supplementation among under-5 children in Nigeria
Source: PLoS One. 2025 Oct 8;20(10):e0315655. doi: 10.1371/journal.pone.0315655 (PMC12507290; doi:10.1371/journal.pone.0315655)
Supplement: Attachment — Submitted filename: Rebuttal letter_10042024.docx [file pone.0315655.s006.docx]

**Response to reviewer’s comments**

We thank the reviewer for reviewing the paper and providing useful feedback. Please see in the table below our responses to each issue raised.

| **Reviewer #1: Comments** | **Authors’ response** |
| --- | --- |
| Thanks for allowing me to review this interesting work. Overall, I think this is important work and worth publishing. However, I think the authors need to reconsider their methods and focus as currently presented.   1. They have compared costs for two different locations and timepoints and for 1 cycle of a 4 or 5 cycle intervention - SMC. I feel like there could be many potential confounders and that this is a very serious limitation. While the authors point this out in the limitations I wonder if they considered broadening their analysis to cover all cycles in the intervention and/or could adjust for any confounders in location or delivery model and timings in the analysis and present this? They compare to other cost studies which show a total intervention cost also so it would be better to reflect the same in their analysis if possible.   Some other specific points:  2. Layout and structure - the whole piece needs to be reviewed and ensure the correct elements are in the correct sections - e.g. currently elements of the discussion around the inclusion and rationale for specific costs are included in the results - there need to be moved to the correct sections 3. Ethics - please add date and reference for approval 4, Readability - please check the text for readability overall - there are a few instances where it doesn't make sense - for example in L209 - this is not a sentence. Formatting - the authors should check text formatting as the fonts etc are not uniform  Happy to review again once these comments are addressed. | 1. We would like clarify that, in terms of locations, the cycles being compared for the cost analysis were carried out in the same locations.   Based on the intervention design, there were 4 cycles of SMC in the study location as at the time of this study. Cycles 1 – 3 were designed to run the same way with the delivery of SMC-only from house-to-house, while cycle 4 was designed to deliver vitamin A in addition to SMC from house-to-house, we don’t have any indication that this was not so. However, to rule out any possible confounding, we included a sensitivity analyses in our result focusing explicitly on critical cost components (distribution, SPAQ, labour, and supplies). These analyses evaluated the robustness of our findings by systematically varying costs within plausible ranges (±10% to ±20%) and assessed their impact on total costs per child. The findings, detailed in Annex 2 of our submission documents, showed moderate sensitivity to changes in distribution costs ($0.03–0.04 per child), low-to-moderate sensitivity to labour and SPAQ costs ($0.01–0.03 per child), and minimal sensitivity to supplies costs.   2. We have revised accordingly in tracked changes and moved discussion elements to the discussion section, where we have provided additional contextual information and interpretation to enhance clarity. 3. We have revised the ethical consideration section accordingly to reflect the data and approval reference number. 4. We apologize for the typographical errors in the document. We have revised accordingly.   Thank you for your useful feedback. |
